# Acute global longitudinal strain evaluation in patients with subacute to chronic chest pain: A pilot, observational study^☆^

**DOI:** 10.1016/j.ahjo.2023.100342

**Published:** 2023-10-31

**Authors:** Paramjit Kaur, Syed Fatmi, Emmanuel Tangco, Elise E. Zhao, Fateeha Tariq, Sanjida Jahan, Kristy Johnson Pich, Darius Aliabadi

**Affiliations:** a Southeast Medical Center, Dothan, AL, Internal Medicine, United States of America; b Carilion Medical Center, Roanoke, VA, Internal Medicine, United States of America; c Southeast Medical Center, Dothan, AL, Internal Medicine GME, United States of America; d Southeast Medical Center, Dothan, AL, Department of Cardiology, United States of America

**Keywords:** 2D strain echocardiography, Cardiac events, Global longitudinal strain, Balloon inflation, Subacute ischemia, Percutaneous coronary intervention

## Abstract

**Background:**

Global longitudinal strain (GLS) imaging is a multifaceted modality that has been utilized in various fields of clinical cardiology in the recent past; however, its implementation for the assessment of ischemia has been limited.

**Objectives:**

This study aimed to document the functional changes in GLS secondary to acute myocardial ischemia in patients with chronic chest pain.

**Methods:**

In this unblinded, single-center, investigator-initiated, prospective pilot study, the functional changes in GLS at baseline, during, and immediately following coronary percutaneous intervention were monitored in 10 ambulatory patients who underwent elective catheterization. The exclusion criteria included a low ejection fraction, or a history of chemoradiation, myopathy, and congenital heart disease.

**Results:**

The average GLS at baseline, during the balloon intervention (BI), and 1–2 min after BI was −15.4 % ± 3.3 %, −10.2 % ± 3.6 %, and −16.1 % ± 4.2 %, respectively. The average GLS decreased significantly by 5.1 % (95 % CI, −7.9 % to −2.3; P = 0.0013) from baseline to BI, increased by 6.3 % (95 % CI, 3.7 % to 8.9 %; P < 0.001) from BI to immediately post-BI, and increased by 0.7 % from baseline to post-BI (95 % CI, −0.4 % to 2.7 %; P = 0.161).

**Conclusion:**

Patients undergoing BI showed a significant decrease in the average GLS within 1–2 min of BI, with GLS returning to baseline subsequently, clearly demonstrating the efficacy of the modality and the clinical significance of data obtained. These functional changes replicate cardiac perfusion to the segments supplied by respective vessels and its effect with reperfusion or ballooning. The slight increase in GLS from baseline to post-intervention was not statistically significant, which could be attributed to the confounding factors. Analyzing our data, we can safely conclude that GLS is potentially a sensitive, temporal, and quantitative tool for identifying patients with acute ischemia with its limitations and need for further perfection of this modality. Therefore, GLS assessments on 2D echo can be used for risk stratification of patients with subacute to chronic chest pain concerning for ischemia in addition to EKG, troponins and other data obtained by non-invasive testing and evaluation.

## Graphical abstract

**Figure:**
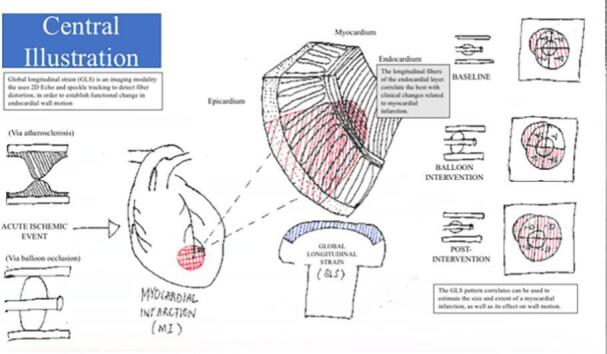

## 1 Introduction

Global longitudinal strain (GLS) measurement is an indispensable modality that utilizes 2D echocardiography and speckle tracking to estimate functional changes in endocardial wall deformation [1].The endocardium is the furthest layer from the coronary circulation, making it the most susceptible layer to acute ischemia and subsequent infarct [2]. Among the three directions of fibers comprising the endocardium, namely the longitudinal, circumferential and radial fibers, it is the longitudinal fibers which have been found to correlate well with clinical changes and measuring longitudinal strain patterns would theoretically correspond with the size and extent of a myocardial infarction [3].

In prior literature, GLS has already been studied in various settings in the evaluation of cardiac function. It has been used in the long-term monitoring of ventricular dysfunction in certain patient populations, such as those with ST-elevation myocardial infarction (STEMI) who have undergone intervention, those who are on potentially cardiotoxic chemotherapy and those who have undergone pacemaker placement [[4], [5], [6]]. Previous studies have also looked into the potential of GLS in augmenting the findings of dobutamine stress testing among patients with coronary artery disease [7,8]. GLS values have also been applied to predicting left ventricular function recovery two to six months after myocardial infarction [9,10]. However, data on the accuracy of GLS and its utility in the setting of acute ischemia and eventual reperfusion are limited.

In the present study, we aimed to explore the utility of GLS measurement in risk stratification for patients with subacute to chronic ischemia undergoing elective left heart catheterization.

## 2 Methods

### 2.1 Ethics

This study involved human participants, and all procedures were followed in accordance with ethical standards on human experimentation with the Institutional Review Board committee.

### 2.2 Study oversight

The study was an investigator-initiated, single-center, unblinded, prospective pilot study. The research protocol was approved by the Institutional Review Board of our institution, a regional referral center. The study was funded by our institution's Department of Cardiology. The investigators did not receive funding from any other institution or company. The authors assume full responsibility for the accuracy and completeness of the data and analysis.

### 2.3 Patient selection

A cohort of 10 patients (male, 4; female, 6; mean age, 72.5 ± 5.9 years) was selected from patients aged ≥18 years who underwent outpatient evaluation for intermittent chest pain at an outpatient cardiology clinic within a 4-month span between March 2022 and July 2022. Patient demographics, including their involved coronary arteries, are detailed in Table 1. These patients have either a negative or an inconclusive result from their most recent stress test and have been scheduled to undergo elective left heart catheterization for further evaluation. Those patients selected to be part of the cohort were screened to have no history of STEMI, history of ischemic cardiomyopathy, exposure to cardiotoxic chemotherapy, prior radiation therapy or any history of congenital heart disease. During catheterization, participants with a low ejection fraction (left ventricular ejection fraction <40 %) and those with poor-quality 2D echocardiogram images were also excluded. All patients provided written informed consent to be included in our pilot study.

**Table 1:** Patient demographic data.

Patient	Sex	Age	Coronary artery
1	M	69	Proximal non-dominant left circumflex artery
2	F	74	Large non-dominant mid-circumflex artery
3	F	69	Diagonal
4	F	72	Proximal large dominant right coronary artery
5	F	71	Mid-PDA
6	M	67	Mid-LAD
7	F	84	Mid-LAD
8	F	78	Distal dominant right coronary artery
9	M	64	Proximal LAD ostium
10	M	67	LAD

LAD, left anterior descending artery; PDA, posterior descending artery.

### 2.4 Procedures

All participants underwent the same intervention. Patients underwent left heart catheterization to identify the stenosed culprit vessel with significant obstruction. Once the stenosis was identified, GLS was assessed with a limited transthoracic 2D echocardiogram and this measurement was designated as the “baseline” measurement. Subsequently, this procedure was repeated during balloon intervention while the coronary vessel was occluded for 1–2 min, and this measurement was designated as the measurement “during balloon intervention”. Finally, another limited echocardiogram with GLS was obtained within 2 min post-intervention, and this was designated as the measurement “post-intervention”.

In order to limit confounding factors, all patients were evaluated using the same 2D echocardiogram machine (Philips Affiniti CVx Cardiovascular Ultrasound System, Philips Medical Systems, Andover, MA) and GLS imaging software (QLAB Advanced Quantification 70 Auto Strain). All studies were performed by the same echocardiogram technician, who was credentialed to perform GLS. All data were collected onto spreadsheets, and their corresponding GLS values and images were analyzed.

### 2.5 Statistical analysis

Statistical analyses were performed using MedCalc Statistical Software version 20.112 (MedCalc Software bv, Ostend, Belgium). Continuous variables were presented as mean ± standard deviation, unless otherwise stated. The GLS at each time point was assessed for normality. The data showed no outliers and were normally distributed at each time point, as assessed by visual inspection of the boxplot and the Shapiro-Wilk test (P > 0.05). A one-way repeated-measures analysis of variance (ANOVA) was conducted to determine whether the GLS obtained at baseline and during and after the intervention showed statistically significant differences [11]. The epsilon (ε) was 0.740, as calculated according to Greenhouse and Geisser [12], and was used to correct the one-way repeated-measures ANOVA. GLS was significantly different at different time points during left heart catheterization (F (1.481, 13.327) = 34.4, P < 0.001) [13]. Post-hoc analysis with Bonferroni adjustment was used to compare the mean differences in GLS between the time points [14].

## 3 Results

**Fig. 1:**
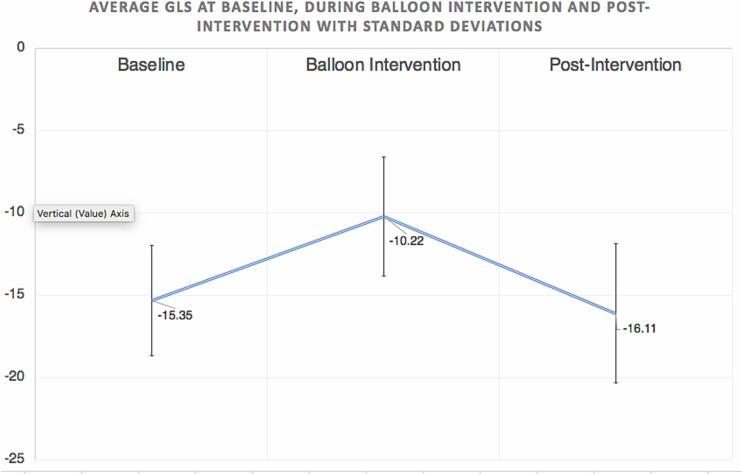
Line graph with the average Global Longitudinal Strain patterns at baseline (during balloon intervention and post-intervention with their corresponding standard deviations. Statistically significant change was noted during balloon intervention while the slight difference between baseline and post-intervention GLS was statistically insignificant.

The average baseline GLS in the cohort of 10 patients was −15.4 % ± 3.3 %. The average GLS during balloon intervention was −10.2 % ± 3.6 %. The average measurement obtained immediately after balloon inflation (post-intervention) was −16.1 % ± 4.2 %. We observed a statistically significant decrease of 5.1 % (95 % CI, −7.9 to −2.3; P = 0.0013) from baseline to balloon intervention and a significant increase of 6.3 % (95 % CI, 3.7 to 8.9; P < 0.001) from balloon inflation to immediately post-intervention. However, while the GLS increased slightly from baseline to post-intervention, the increase was not statistically significant (0.7 %; 95 % CI, −0.4 to 2.7; P = 0.161). These results are summarized in Fig. 1. No outliers were noted, and the data were normally distributed at each time point in the analysis with a statistically significant P value.

## 4 Discussion

To our knowledge, our observational study is the first to look into the hyperacute to acute changes that occur during ischemic events. A porcine model study by Howard-Quijano et al., which performed longitudinal strain measurements within 15 min of coronary artery occlusion, had observed that longitudinal strain was sensitive to ischemia and that this had diagnostic potential in the evaluation of acute ischemia [15]. Our study measured a statistically significant change in GLS during and after an acute coronary artery occlusion. We were able to appreciate GLS changes within 2 min of an ischemic event, which suggest that there is utility in considering point-of-care GLS measurement in the evaluation of acute coronary syndrome. Though this may prove impractical in clinical practice due to the need for trained technicians, these findings correlate well with a previous study by Norum et al. that looked into the use of GLS measurements in the emergency department setting. Their study established sensitive and specific cutoff values for GLS changes which helped identify coronary artery disease in patients who presented without elevated troponins [16]. Beyond the potential for improving point-of-care evaluation, the results of our study could also point towards GLS being useful in evaluating for the need of elective heart catheterization in patients with subacute to chronic chest pain.

Our study was also able to detect an improvement between the pre-intervention baseline and post-intervention GLS measurements, but at a higher P value (P = 0.161). Increasing the power of future studies could verify the utility of GLS in monitoring ventricular recovery after an acute coronary event and reperfusion. In a study by Cimino et al., where GLS changes where monitored within a six day interval in patients with STEMI who received timely interventions, they determined GLS changes to be sensitive and specific in monitoring global myocardial function and detecting transmural necrosis [4].

As an additional point of discussion, the apical region was always observed to be affected first during catheterization and subsequent coronary occlusion. This observation was consistent in all ten of our patients, highlighting the variance of the blood supply of the apical region as well as its anatomical susceptibility to ischemia, regardless of the culprit vessel undergoing intervention. This information can also contribute to potential utility in point-of-care GLS evaluation of an index event because certain disease entities, such as Takotsubo cardiomyopathy, have been shown in emerging literature to have a pathognomonic GLS pattern, different from the apical patterns we saw in our cohort [17].

## 5 Limitations

**Fig. 2:**
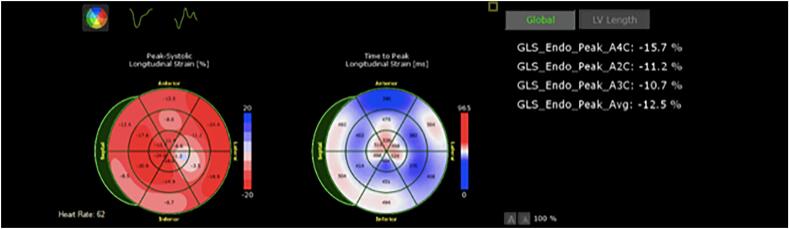
A sample set of GLS measurements in one of our patients (Patient 7) demonstrates the detectable change during balloon intervention and subsequent return to GLS pattern comparable to baseline. This figure corresponds to the baseline.

**Fig. 3:**
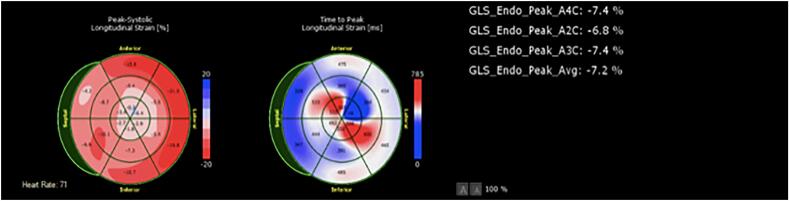
A sample set of GLS measurements in one of our patients (Patient 7) demonstrates the detectable change during balloon intervention and subsequent return to GLS pattern comparable to baseline. This figure corresponds to the GLS pattern during balloon intervention.

**Fig. 4:**
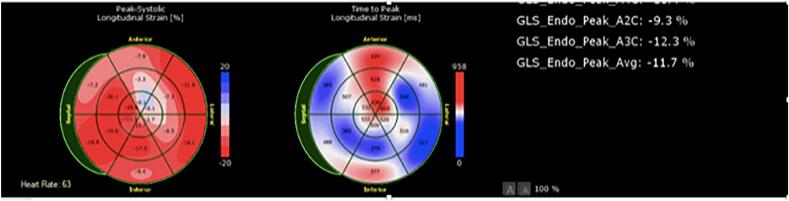
A sample set of GLS measurements in one of our patients (Patient 7) demonstrates the detectable change during balloon intervention and subsequent return to GLS pattern comparable to baseline. This figure represents the post-intervention GLS.

Our sample size was small. Furthermore, we excluded patients who have had prior myocardial infarctions, a history of chemoradiation, a history of cardiomyopathy and those with low ejection fractions, which may limit our generalizability to patient with coronary artery disease, as these are common co-morbidities. Direction of future research can look into including these co-morbidities and risk factors so that stronger and more representative data on the utility of GLS in point-of-care or outpatient evaluation for patients with chest pain can be generated. It can also be noted that although our patients showed immediate detectable measurements in GLS, our study did not track measurements beyond our two-minute timeframe (Fig. 2, Fig. 3, Fig. 4).

## Clinical perspectives

Our study data showed that GLS is potentially a sensitive, temporal, and quantitative tool for detecting ischemic changes among patients with subacute to chronic chest pain. Within our study, GLS changes were noted to be detectable within 2 min of an ischemic event. These measurements can be used to improve risk stratification of patients with inconclusive outpatient workup, to help determine the need for any further invasive evaluations. GLS can also potentially be used in point-of-care evaluation of acute chest pain, as GLS has been known to complement troponin evaluations and certain disease entities can have specific GLS patterns.

## Funding disclosure

None.

## Declaration of competing interest

The authors declare that they have no known competing financial interests or personal relationships that could have appeared to influence the work reported in this paper.
